# Supplementary material for: Acetyl-DL-leucine in two individuals with REM sleep behavior disorder improves symptoms, reverses loss of striatal dopamine-transporter binding and stabilizes pathological metabolic brain pattern—case reports
Source: Nat Commun. 2024 Sep 2;15:7619. doi: 10.1038/s41467-024-51502-7 (PMC11369233; doi:10.1038/s41467-024-51502-7)
Supplement: Supplementary file 1 — Supplementary Information [file 41467_2024_51502_MOESM1_ESM.pdf]

NCOMMS-24-10685-B

Supplementary Information

Oertel et al. Nature Communications

The Supplementary Information provides additional information on

A) Acetyl-DL-Leucine (ADLL)

B) Information on Clinical Data

  B1) Supplementary Table 1 - Time schedule of DAT-SPECT and FDG-PET imaging and related results of clinical evaluations for patient 1 and patient 2.

  B2) Supplementary Table 2 - Subjective assessment of the effect of ADLL on the RBD phenotype

C) The therapy-responsive clinical outcome measure "3-week RBD-severity sum-score (RBD-SS-3)"

D) Details of the DAT-SPECT procedure, the olfactory function test and MIBG scintigraphy

E) Limitations

F) References of the Supplementary Information

A) Acetyl-DL-Leucine – additional information

Availability – safety and tolerability

The commercially available drug Tanganil™ contains the racemate acetyl-DL-leucine. Only the L-enantiomer of acetyl-leucine is bioactive. Each tablet contains 500 mg acetyl-leucine (250 mg of the active enantiomer acetyl-L-leucine and 250 mg of the inactive enantiomer acetyl-D-leucine). Isolated acetyl-L-leucine is not available as therapy. The patent for acetyl-DL-leucine, acetyl-D-leucine and acetyl-L-leucine in the indication "treatment of neurodegenerative disorders" lies with the company IntraBio in London, UK.

Acetyl-DL-leucine has been sold to thousands of patients for the indication "vertigo" for >60 years. No minor or major side effect was reported in the annual reports on adverse effects. In summary, the drug is considered to possess a very high tolerability and safety profile.

Note of caution: the commercially available drug ADLL (Tanganil®) contains the substance gluten as a filling component. Individuals with a gluten-intolerance should not take the compound.

Administration, mode of action – further supportive published evidence

ADLL is administered orally and taken up by the ubiquitously expressed monocarboxylate transporters, thereby delivering ADLL to all tissues including the central nervous system[1,2]. AL may increase the production of ATP by modulating the function of pyruvate dehydrogenase. It also has a beneficial effect on impaired lysosomal mechanisms[3–5]. Other work reports an improvement of membrane function with ADLL exposure[6,7]. Two recent methodically different studies support a potential role of N-acetyl-L-leucine (ALL) and leucine in PD: First, in a toxin (MPTP)-mouse model of PD, it was demonstrated that ALL protects MPTP-treated mice by suppressing Desulfobacterota via the gut-brain axis. It was also found that oral administration of ALL alleviated MPTP-induced dopamine neuronal deficits and motor impairments[8]. Second, a two-sample Mendelian randomization study (37,688 patients with PD, 18,618 proxy cases, and 1,417,791 controls) found that an increase of circulating leucine levels decreases PD risk to 63% per standard deviation ([95% CI] = 0.628 [0.467, 0.843]) ; FDR = 0.021)[9].

Individual case of off-label use – compassionate use

Two subjects with iRBD, who had been in our care for 9 (patient 1) and 2 years (patient 2), respectively, were selected. For any emergency situation, the two patients had the private home phone and mobile phone number of the principal investigator (WHO).

The 2 patients received a detailed explanation and information about the nature of a compassionate use (individual case of off-label use) of a compound which is not licensed in Germany at all, and not licensed for the indication “RBD” in France, the only country in which ADLL is commercially available (see above). This information was presented by the first author orally and in writing. The information contained all available information about ADLL in respect to potential side effects, dosage, and

experience with ADLL in the approved indication "vertigo". The information, however, did not contain any details about:

- the predicted latency to the onset of a potential effect of ADLL or
- the duration of a potential effect of ADLL on the clinical phenotype RBD, in case the patient stopped the treatment on her/his own or in case a withdrawal was recommended.

These steps were taken in order to keep the patient blinded at least for these two aspects of the ADLL-treatment.

The patient consent form was signed by the patient and the informing physician (WHO) and the document was kept in a safe with access restricted to two people (AJ, WHO).

Both iRBD patients also participated in a long-term 10-year natural history study called REMPET[10,11]. This study includes serial FDG-PET imaging every 3 to 4 years, a baseline ^{123}I-MIBG scintigraphy, a baseline DAT-SPECT and subsequent DAT-SPECT scans and a baseline Sniffin Sticks test for olfactory function.

Furthermore, participants undergo several other clinical investigations including UPDRS-III[12], MoCA[13] and SCOPA-AUT[14] at least annually and in some cases every 6 months. The approval of the local ethics committee for the REMPET-study (Faculty of Medicine, University of Marburg: document 49/16, 20. April 2016) is available on request.

B) Clinical information

B1) Supplementary Table 1 | Time schedule of DAT-SPECT and FDG-PET imaging and related results of clinical evaluations for patient 1 and patient 2.

This information is also illustrated in Figure 2 in the Main Manuscript.

**Patient 1**						
**Date**	**Imaging performed**	**UPDRS I-III / MDS-UPDRS I-III**	**UPDRS III / MDS-UPDRS III motor function w/o action tremor**	**MoCA cognitive function**	**TDI olfactory function**	**MIBG <1.5 patho-logical**
February 2013	DAT-SPECT	UPDRS 4	2	20	16.00	–
January 2014	FDG-PET	UPDRS 6	4	28	15.50	–
March 2014	DAT-SPECT	UPDRS 6	4	28	–	–
May 2014	MIBG	–	–	28	–	1.32
March 2015	-	UPDRS 8	3	27	10.00	–
April 2016	DAT-SPECT	UPDRS 5	2	28	12.00	–
August 2018	FDG-PET	UPDRS 6	3	–	11.50	–
January 2019	DAT-SPECT	UPDRS 4	3	30	6.00	–
November 2021	***Start of ADLL Therapy***	MDS-UPDRS 11	4	30	5.00	–
February 2022	DAT-SPECT	–	–	–	10.00	–
December 2022	FDG-PET	MDS-UPDRS 10	4	28	4.00	–
September 2023	DAT-SPECT	MDS-UPDRS 11	2	25	10.00	–
**Patient 2**						
**Date**	**Imaging performed**	**MDS-UPDRS I-III**	**MDS-UPDRS III motor function w/o action tremor**	**MoCA cognitive function**	**TDI olfactory function**	**MIBG <1.5 patho-logical**
August 2020	DAT-SPECT	1	0	29	15.50	–
June 2021	FDG-PET	0	0	29	11.00	–
January 2022	***Start of ADLL therapy***	3	0	30	10.00	–
May 2022	MIBG	–	–	–	17.25	0.98
January 2023	FDG-PET	2	0	29	13.50	–
July 2023	DAT-SPECT	0	0	27	13.25	–

B2) Supplementary Table 2 | Subjective assessment of the effect of ADLL on RBD phenotype

	Patient	Patient 2
Did you have severe RBD phenotype with violent movements and aggressive dreams before the therapy with ADLL?	Yes	Yes
Are these severe stages of RBD present under ADLL therapy?	Following therapy with ADLL, the aggressive dream content and the severe movements became less every month and now are nearly absent.	Under ADLL therapy the severe stages of RBD are absent.
Is the frequency the same, did the frequency increase or decrease?	Very much decreased	Very much decreased
Which stages of severity do you or your partner notice under ADLL treatment?	Most nights I have no events during the week.<br>If the RBD symptoms occur, they are milder than before the therapy with ADLL. It happens in general one or two nights out of 7 nights, and I just talk. Occasionally, I move my arms and/or legs, very rarely a bit violently.	Most nights, I have no events during a week.<br>If the RBD symptoms occur, this happens in general one out of 7 nights, and I just talk.<br>Very rarely I move my arms and/or legs, but not violently.
Is the content of your dreams aggressive? Please describe the quality of your dream content.	Aggressive dreams are completely absent. If I have dreams, they are of pleasant content. Very rarely I defend myself against someone who frightens me. In the vast majority, I cannot remember any dreams anymore.	Aggressive dreams are completely absent. If I have dreams, I escape an unpleasant situation or defend myself against someone who slightly frightens me.
Did you notice any particular trigger to induce RBD phenotype?	Very strong emotional situations (patient does not drink alcohol)	Alcohol intake (during holidays), marked change in daily rhythm
Did the severity and frequency of the RBD symptoms change in the last 6 months?	No	Over the last 4 months nearly absent – one single night with slightly violent movements of extremities. I do not know why. I could not identify a trigger.
Did you change your previous medication for RBD?	Yes, I have withdrawn clonazepam for more than 14 months. I do not need it anymore.	No, I had no previous mediation
Any other comment?	Constipation markedly improved	Questionable improvement in smell function
Adverse effects	None	Initially slight weight loss, was reversible

C) The therapy-responsive clinical outcome measure

"3-week RBD-severity sum-score" (RBD-SS-3)

Daily RBD-severity scores (daily RBD_SS) of three consecutive weeks were added to create a 3-week RBD severity sum-score (RBD-SS-3 – score range from 0 to 84 with 84 indicating the highest degree of severity). The values of the RBD-SS-3 are presented in the main manuscript in Figure 1a (Patient 1) and Figure 1b (Patient 2) for the first 18 weeks and the last 18 weeks of the observation period of 83 weeks.

This mode of presentation provides 6 consecutive values for the first 18 weeks under ADLL-therapy and demonstrates the delayed onset of the effect of ADLL in week 4. It also provides 6 values for the last 18 weeks under ADLL-therapy and demonstrates the persistence of the reduction in the severity of the RBD phenotype for the weeks 65 to 83.

Patient 1 – reported outcome

Supplementary Figure 1a presents the daily RBD-SS of patient 1 in the first 18 weeks (124 days) and the last 18 weeks of the treatment period of 581 days. The figure allows assessment of the frequency and severity of the RBD events at a daily base. At the beginning of week 4, a marked improvement of the daily RBD-SS was observed.

Withdrawal of symptomatic therapy with clonazepam possible

At week 13 of the ADLL-treatment phase, it was decided to attempt a withdrawal of the symptomatic therapy clonazepam (1 mg at night). Clonazepam was slowly tapered off over 4 days. No change in RBD severity was observed over the next 9 days. In week 15, i.e. on day 14 of the clonazepam withdrawal, patient 1 participated in an event with an extremely high emotional burden. The two following nights patient 1 experienced RBD symptoms of severity level 2. On the following day, it was decided to increase the dose of ADLL from 2-2-5 (4.5 g ADLL) to 2-2-7 (5.5 g ADLL) tablets. The following nights, no RBD event was noticed by patient 1 and her husband. The slightly increased dose of ADLL was maintained for the rest of the treatment period, but the last dose of 7 tablets was split into 3 tablets and one hour later 4 tablets to improve the compliance of the intake of ADLL. Over the whole following treatment period with ADLL, patient 1 did not request additional symptomatic therapy: neither the reinstatement of the clonazepam therapy nor therapy with melatonin. During the following months of ADLL-therapy, patient 1 occasionally experienced a single event (one night) with a daily RBD severity score of maximally 2.

Thus, in summary, the reduction of the severity of the RBD phenotype was maintained under ADLL-therapy and in addition, symptomatic therapy (i.e. clonazepam) could be withdrawn without a subsequent noticeable persistent increase of the severity of the RBD phenotype.

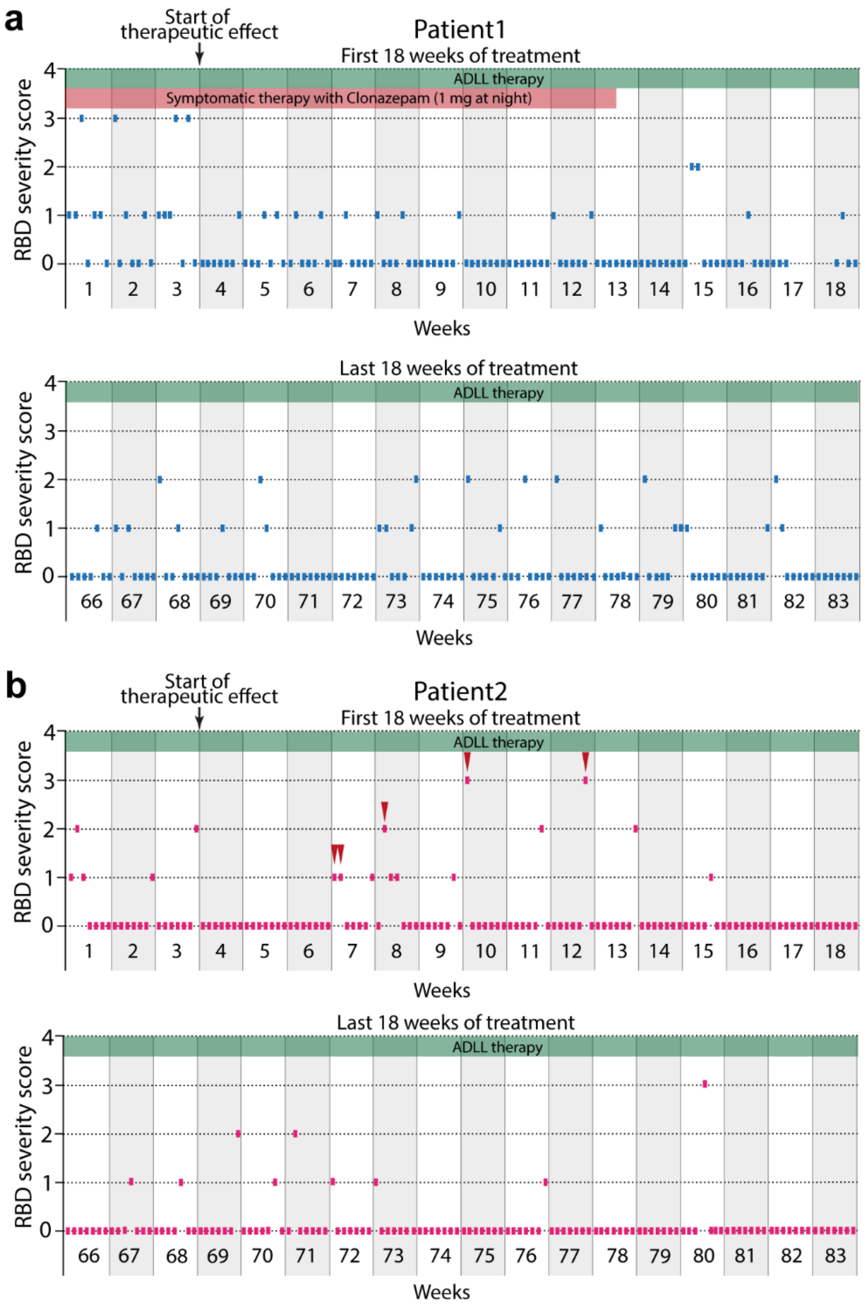

Supplementary Figure 1 | Daily scoring of the severity and frequency of RBD symptoms.

a. Daily RBD severity scores of patient 1 as reported by the patient and spouse. Upper graph represents the first 18 weeks of ADLL-treatment (from 6th November 2021 to 9th March 2022), the lower graph the last 18 weeks of ADLL-treatment (from 2nd February 2023 to 9th June 2023). Patient 1 received symptomatic treatment of RBD

with clonazepam (1 mg/day) until week 13 of ADLL-treatment. Hereafter, clonazepam was withdrawn over a period of 4 days without adverse events.

b. Daily RBD severity scores of patient 2 as reported by the patient and spouse. Upper graph represents the first 18 weeks of ADLL-treatment (from 24th January 2022 to 27th May 2022), the lower graph the last 18 weeks of ADLL-treatment (from 15th May 2023 to 18th September 2023). Red arrowheads indicate alcohol consumption.

Patient 2 – reported outcome

Supplementary Figure 1b presents the daily RBD-SS of patient 2 in the first 18 weeks (124 days) and the last 18 weeks of the treatment period of 581 days. The figure allows assessment of the frequency and severity of the RBD events at a daily base. At the beginning of week 4, a marked improvement of the daily RBD severity score was observed for the next three weeks. In weeks 7 to 12, the patient noticed a recurrence of severe RBD symptoms on a few nights (see Clinical Information – Figure 2b (main manuscript) – paragraph on alcohol and severity of RBD phenotype (see below)).

During the last 18 weeks of ADLL-therapy, patient 2 experienced two single nights with a daily RBD-severity score of 2 and a single night with a score of 3 (week 80). This score value of 3 was considered by patient and spouse as lying between 2 and 3. For the Supplementary Figure 1b the value was noted as score 3, as all values were marked against the working hypothesis, i.e., not as 2 - 3, but as 3.

Effect of substantial amount of alcohol on severity of RBD phenotype under ADLL-therapy

During weeks 7, 8, 10, and 12, the patient went on short vacations or took part in social events. During these vacations/events, he changed his diurnal rhythm, went to bed later than usual and consumed a substantial amount of alcohol in the late evening. Each time following substantial alcohol consumption, he noticed the occurrence of marked RBD severity (level 1, 2 and 3 – see red arrows in Figure 1b in main manuscript and in Supplementary Figure 1b) in the subsequent night. In week 12, it was obvious that the reoccurrence of RBD events was related to alcohol consumption. It was therefore decided that he would stop consuming any alcohol during the time he was taking ADLL. To our surprise, neither the patient himself nor his spouse had previously ever made the connection between RBD

events and alcohol consumption. On careful taking of the history of alcohol intake, we established that he had not consumed any alcohol in the first six weeks under ADLL-therapy. Thus, the values of the daily RBD-SS of the first six weeks are not confounded by alcohol intake. The fact that alcohol intake can induce RBD or aggravate the severity of the RBD phenotype – especially in men – has repeatedly been reported[15].

D) Details on the DAT-SPECT procedure, the olfactory function test and on [^{123}I]-MIBG scintigraphy

DAT-SPECT procedure

Briefly, after blocking the thyroid gland with sodium perchlorate, 185 MBq ^{123}I-FP-CIT (GE Healthcare) was administered to the patient intravenously. Scans were acquired on a dual-headed gamma camera (Symbia S; Siemens, Erlangen, Germany) exactly 180 minutes after injection. The images were obtained in 120 projections over a 360° arc with the step-and-shoot mode (40 seconds per projection). The quality of the DAT-SPECT procedure was independently and repeatedly controlled by the imaging group of the Parkinson’s Progression Marker´s Initiative study of the Michael J Fox Foundation, New York USA.

Olfactory function test

Threshold subtest: the tested subject first learned to identify the odor n-butanol at the highest concentration tested by exposure to the pen with the highest concentration. Subsequently, the subject was exposed repeatedly to three pens: two blanks and one with the odor n-butanol in 16 different concentrations (1 = highest concentration, 16 = lowest concentration). During the test, the so-called turning point was determined seven times; this corresponds to the highest odor dilution that was correctly detected by the patient twice in one run. The threshold (T) score was the average value of the dilution steps of the last four turning points (range 1–16).

Discrimination subtest: three pens – two with the same and one with a different odor – were presented. The subject always had to identify the one pen that smelled different. This task was

repeated 16 times in 30 s time intervals. The discrimination (D) score was the sum of correct answers ranging from 0–16.

Identification subtest: subjects were presented with 16 different odors and were asked to identify these from a given choice of four possibilities. The number of correct answers resulted in the identification (I) score (range 0–16).

The full range Sniffin Sticks test is very demanding and takes at least one hour to complete. The result depends on the level of concentration and honesty of the participant. Despite careful instructions, in our experience severely anosmic patients have a tendency to guess the odor instead of stating ”I am not able to identify the odor”. Patient 1 was markedly anosmic just before the initiation of ADLL-therapy. There is a small increase in the TDI sum-score (see Figure 2a in the Main Manuscript). When asked whether the patient noticed any improvement in smell function over the course of the ADLL therapy she stated NO. In contrast, patient 2 was less impaired – although formally fulfilling the criteria of anosmia. After a few months of ADLL therapy he unsolicitedly stated that he had the impression that his smell function had slightly improved. The TDI scores improved from 10 (before initiation of ADLL-therapy) to 17 under ADLL-therapy and then dropped down again to 13 (anosmic) (still under ADLL-therapy - see Figure 2b in the Main Manuscript). In summary, the long term therapy with ADLL failed to change the anosmic status of the two IRBD patients. Therefore, we consider it unjustified to discuss the TDI sum-score changes in patient 2 as a potential sign for an improvement of olfactory function. This issue remains to be studied in RBD patients preferentially with a TDI sum-score of 16 and higher (hyposmia) at baseline in order to increase the likelihood to observe a beneficial effect of ADLL on the non-motor symptom “olfactory impairment”.

[^{123}I]-Metaiodobenzylguanidine scintigraphy

After blocking of the gastric and thyroid sodium iodide symporters, images were acquired 4 hours after injection of 185 MBq (±10%) [^{123}I]MIBG (AdreView Iobenguane, GE Healthcare, Braunschweig,

Germany) using a dual-head gamma camera with a low energy high-resolution collimator (Siemens, Symbia, Erlangen, Germany) at a window setting of 159 keV (±10%).

E) Limitations

Low number of investigated iRBD patients

This case report was carried out according to the rules of compassionate use in Germany. For subjects without obvious handicap and a non-lethal disorder – as is the case in iRBD – it is recommended to keep the number of participants in a compassionate use study (see above – Compassionate use) at three or lower. In addition, investigations not related to the safety of the participants are to be kept to a minimum. This framework restricts the choice of subjects. We therefore included two patients with iRBD, who participated in a long-term natural history study on iRBD called REMPET[10,11,16]. In this study, participants undergo baseline MIBG-scintigraphy and serial investigations with DAT-SPECT and FDG-PET. Thus, additional imaging investigations with exposure to a radioactive ligand could be kept to a minimum during the therapy with ADLL.

Unblinded study

This case report was not blinded. However, as this is the first time that the compound ADLL has been administered to individuals suffering from iRBD, neither the principal investigator nor the patients had any idea about the degree of effect to expect and at what time-point an initial effect of ADDL might be observed.

Although two of the authors (WHO, MS) knew from the index PD patient (see Introduction of the main manuscript) that an effect on the RBD phenotype might be expected after about 5 weeks, it was not possible to predict whether the subjects with iRBD would experience the same latency, if at all. In fact – and unexpectedly – the effect on the RBD severity sum-score appeared already after three weeks of ADLL therapy in both iRBD patients.

Furthermore – as stated below –, the two participants were informed that the compound ADLL might improve their RBD phenotype, but they did not receive any information on whether this improvement would be symptomatic or based on a disease-modifying effect or whether an effect would be related to the dream content or the dream enactment. In addition, they were not informed that the effect of ADLL in the index PD patient had been obvious after a delay of 5 weeks. Thus, from a pragmatic – but not from a formal – point of view both patients were somehow blind.

Clinical outcome measure – patient-centered subjective RBD phenotype severity rating with an RBD diary

First, the recording of the RBD-severity score is subjective and reflects the impression of the patient and spouse. Thus, the RBD-diary provides patient-centered data. The RBD-diary itself has not been validated in a multicenter study[17]. Furthermore, if the RBD event was not strong enough to wake up the spouse, mild RBD events (severity level 1, may be even severity level 2) were missed. And we are aware that the quality of sleep of the spouse substantially contributes to whether the spouse notices RBD symptoms of the patient. Thus, the documentation of the RBD-severity and frequency most likely underrepresents the frequency and severity of the RBD phenotype established before and under effective ADLL-therapy. From the perspective of the patient and spouse, however, this is not important, as the disturbing feature of RBD in general is the disruption of sleep by severe RBD symptoms (shouting, hitting) and the spouse's fear of being injured by the patient due to violent dream enactment of the partner.

To partially overcome this limitation, we captured the overall assessment of the effect of the ADLL-therapy by the patient. We asked both patients to answer a list of questions related to the frequency and severity of their RBD phenotype. The questions and the respective answers are presented in the Supplementary Table 2.

The gold standard for an objective assessment of the RBD phenotype is video-assisted polysomnography (vPSG). This method is time-consuming, expert-dependent, not available on a daily

basis and costly, as vPSG requires an in-house recording overnight in a certified sleep laboratory. These features restrict the recording of the RBD phenotype to a low number of visits per year. In addition, vPSG does not provide direct information on the dream content.

On the other hand, the availability of instruments to measure a therapeutic effect of a compound on the severity of the RBD-phenotype is limited. We therefore decided to use a recently published RBD diary[17] and modified it slightly. This diary is patient-centered, as it not only scores movements (which can be EMG-recorded in the vPSG during REM-phase), but it also covers the type of dream content. According to current knowledge, aggressive dream content leads to enactment of aggressive behavior. Reduction or disappearance of aggressive dream content can only be assessed by the patient and indirectly by the spouse.

In addition, the atonia or loss of atonia during the REM-dream phase of sleep is controlled by a complex circuitry of nuclei[18]. Thus, we had to consider that the used compound ADLL might change only one aspect of the RBD phenotype, for example the dream content, and would not entirely abolish all movements during REM sleep. We therefore decided not to rely on repeated vPSG, but on the "continuous" daily scoring of the severity of the RBD phenotype by patient and spouse. This daily scoring of the events at night reflects whether a compound provides a benefit for the patient and spouse and is thus a clinically relevant outcome measure.

Lack of a treatment-free baseline

Based on the statement of the index Parkinson patient (see introduction of the main manuscript), who in addition to PD suffered from RLS and RBD, we assumed that – in analogy – the onset of the ADLL effect on the RBD severity would be at week 5 or 6 in patients with isolated RBD. We therefore assumed that we would have a period of 5 weeks to establish a baseline score of the RBD severity and did not perform a run-in period without treatment.

The results of the 2 iRBD cases showed that the decrease of the RBD severity started at the beginning of week 4. Therefore, our baseline value of the 3-week RBD severity score (RBD-SS-3) was

calculated from the first 21 daily RBD-severity scores already under ADLL-therapy. This is a limitation of the study, as we do not have a true baseline score without ADLL-treatment. Nevertheless, the data show that there is a marked difference between the severity and frequency of RBD-events in the first 3 weeks and in all other weeks of the >18 months observation period (see Figure 1 in the main manuscript).

Lack of protocol defined scheduled dates for imaging

To keep the number of additional investigations as low as possible, we adapted the dates of the investigations for DAT-SPECT and FDG-PET to the protocol requirements of the REMPET study. For example, in the case of patient 1, an FDG-PET in December 2022 – 12 months after initiation of ADLL-therapy and under continuous ADLL-therapy – was performed according to schedule of the REMPET-study. However, the respective DAT-SPECT investigation planned in January 2023 – 13 months after the initiation of ADLL-therapy and under continuous ADLL-therapy – was not possible, as the patient had to undergo a medical therapy unrelated to RBD. We therefore postponed the next DAT-SPECT investigation in patient 1 to the two-year time point (22 months) after the initiation of ADLL-therapy.

F) Reference list - Supplementary Information

1. Churchill, G. C., Strupp, M., Galione, A. & Platt, F. M. PloS one 15, e0229585; 10.1371/journal.pone.0229585 (2020).
2. Churchill, G. C. *et al.*. *Scientific reports* **11,** 15812; 10.1038/s41598-021-95255-5 (2021).
3. Bremova-Ertl, T. *et al. Journal of neurology* **269,** 1651–1662; 10.1007/s00415-021-10717-0 (2022).
4. Kaya, E. *et al. Brain communications* **3,** fcaa148; 10.1093/braincomms/fcaa148 (2021).
5. Kaya, E. *et al. Journal of clinical medicine* **9**; 10.3390/jcm9041050 (2020).
6. Vibert, N. & Vidal, P. P. *The European journal of neuroscience* **13,** 735–748; 10.1046/j.0953-816x.2000.01447.x (2001).
7. Hegdekar, N., Lipinski, M. M. & Sarkar, C. *Scientific reports* **11,** 9249; 10.1038/s41598-021-88693-8 (2021).
8. Xu, Z. *et al. Brain research bulletin* **202,** 110729; 10.1016/j.brainresbull.2023.110729 (2023).
9. Cheng, J.-Y., Deng, Y.-T. & Yu, J.-T. *Journal of neurochemistry* **166,** 972–981; 10.1111/jnc.15937 (2023).

10. Meles, S. K. *et al. Movement disorders : official journal of the Movement Disorder Society* **32,** 1482–1486; 10.1002/mds.27094 (2017).

11. Kogan, R. V. *et al. Movement disorders : official journal of the Movement Disorder Society* **36,** 230–235; 10.1002/mds.28260 (2021).

12. The Unified Parkinson's Disease Rating Scale (UPDRS): status and recommendations. *Movement disorders : official journal of the Movement Disorder Society* **18,** 738–750; 10.1002/mds.10473 (2003).

13. Gagnon, J.-F., Postuma, R. B., Joncas, S., Desjardins, C. & Latreille, V. *Movement disorders : official journal of the Movement Disorder Society* **25,** 936–940; 10.1002/mds.23079 (2010).

14. Visser, M., Marinus, J., Stiggelbout, A. M. & van Hilten, J. J. *Movement disorders : official journal of the Movement Disorder Society* **19,** 1306–1312; 10.1002/mds.20153 (2004).

15. Jun, J.-S. *et al. Nature and science of sleep* **14,** 1713–1720; 10.2147/NSS.S372823 (2022).

16. Janzen, A. *et al. Movement disorders : official journal of the Movement Disorder Society* **37,** 624–629; 10.1002/mds.28859 (2022).

17. Kunz, D., Stotz, S. & Bes, F. *Journal of pineal research* **71,** e12759; 10.1111/jpi.12759 (2021).

18. Peever, J., Luppi, P.-H. & Montplaisir, J. *Trends in neurosciences* **37,** 279–288; 10.1016/j.tins.2014.02.009 (2014).
