# Supplementary material for: Acetyl-DL-leucine in two individuals with REM sleep behavior disorder improves symptoms, reverses loss of striatal dopamine-transporter binding and stabilizes pathological metabolic brain pattern—case reports
Source: Nat Commun. 2024 Sep 2;15:7619. doi: 10.1038/s41467-024-51502-7 (PMC11369233; doi:10.1038/s41467-024-51502-7)
Supplement: Supplementary file 3 — Reporting Summary [file 41467_2024_51502_MOESM3_ESM.pdf]

nature portfolio

Corresponding author(s): Wolfgang H Oertel
oertelw@med.uni-marburg.de

Last updated by author(s): Jul 11, 2024

Reporting Summary

Nature Portfolio wishes to improve the reproducibility of the work that we publish. This form provides structure for consistency and transparency in reporting. For further information on Nature Portfolio policies, see our Editorial Policies and the Editorial Policy Checklist.

Statistics

For all statistical analyses, confirm that the following items are present in the figure legend, table legend, main text, or Methods section.

n/a	Confirmed	
☐	☒	The exact sample size (*n*) for each experimental group/condition, given as a discrete number and unit of measurement
☐	☒	A statement on whether measurements were taken from distinct samples or whether the same sample was measured repeatedly
☒	☐	The statistical test(s) used AND whether they are one- or two-sided<br>*Only common tests should be described solely by name; describe more complex techniques in the Methods section.*
☐	☒	A description of all covariates tested
☒	☐	A description of any assumptions or corrections, such as tests of normality and adjustment for multiple comparisons
☐	☒	A full description of the statistical parameters including central tendency (e.g. means) or other basic estimates (e.g. regression coefficient) AND variation (e.g. standard deviation) or associated estimates of uncertainty (e.g. confidence intervals)
☒	☐	For null hypothesis testing, the test statistic (e.g. *F*, *t*, *r*) with confidence intervals, effect sizes, degrees of freedom and *P* value noted<br>*Give P values as exact values whenever suitable.*
☒	☐	For Bayesian analysis, information on the choice of priors and Markov chain Monte Carlo settings
☒	☐	For hierarchical and complex designs, identification of the appropriate level for tests and full reporting of outcomes
☒	☐	Estimates of effect sizes (e.g. Cohen's *d*, Pearson's *r*), indicating how they were calculated

Our web collection on statistics for biologists contains articles on many of the points above.

Software and code

Policy information about availability of computer code

Data collection	Severity of REM sleep behavior disorder phenotype was selfrated by patient and spouse at a daily rate. Clinical evaluation was performed with validated questionnaires by an experienced clinical scientist (study physician). FDG-PET images were aquired in Groningen using a Siemens Biograph mCT64 or mCT40 PET/CT camera and in Marburg using a Siemens Biograph 6 (both (Siemens, Munich, Germany); DAT SPECT images and MIBG scans were aquired using a dual-headed gamma camera (Symbia S; Siemens, Erlangen, Germany); olfactory testing was performed with Sniffin Sticks (Burghart Messtechnik, Holm, Germany)
Data analysis	FDG PET images were spatially normalized to an FDG-PET template in Montreal Neurological Institute brain space using SPM12 software (Wellcome Centre for Human Neuroimaging, London, UK) implemented in MATLAB (version R2019a; MathWorks, Natick, MA, USA), FDG PET patterns were analysed with the "Multivariate - Scaled Subprofile Model / Principle Component Analysis (SSM/PCA)"; DAT SPECT scans were analysed using the Brain Registration & Analysis Software Suite (BRASS; HERMES Medical, Sweden).

For manuscripts utilizing custom algorithms or software that are central to the research but not yet described in published literature, software must be made available to editors and reviewers. We strongly encourage code deposition in a community repository (e.g. GitHub). See the Nature Portfolio guidelines for submitting code & software for further information.

Data

Policy information about availability of data

All manuscripts must include a data availability statement. This statement should provide the following information, where applicable:

- Accession codes, unique identifiers, or web links for publicly available datasets
- A description of any restrictions on data availability
- For clinical datasets or third party data, please ensure that the statement adheres to our policy

All data necessary for the conclusions of the study are available in the main text, tables, figures and figure legends and in the supplementary information Original raw data of the Figures and Tables in the Main Manuscript and in the Supplementary Information are available in the Source Data file.

Research involving human participants, their data, or biological material

Policy information about studies with human participants or human data. See also policy information about sex, gender (identity/presentation), and sexual orientation and race, ethnicity and racism.

Reporting on sex and gender	yes, this is a two case report on one female and one male subject . Sex is determined according to selfreporting and report by the spouse.
Reporting on race, ethnicity, or other socially relevant groupings	N/A
Population characteristics	N/A
Recruitment	The two participants were under medical care of the principle investigator. The two subjects also participated in a long term natural history study on REM Sleep Behavior Disorder, termed REMPET. Apart from isolated REM Sleep Behavior Disorder they were healthy and agreed to take the test compound under individual case of off-label use rules. There was no bias in the recruitment procedure. Written informed consent was obtained.
Ethics oversight	N/A see accompanying letter to the Senior Editor

Note that full information on the approval of the study protocol must also be provided in the manuscript.

Field-specific reporting

Please select the one below that is the best fit for your research. If you are not sure, read the appropriate sections before making your selection.

[x] Life sciences [ ] Behavioural & social sciences [ ] Ecological, evolutionary & environmental sciences

For a reference copy of the document with all sections, see nature.com/documents/nr-reporting-summary-flat.pdf

Life sciences study design

All studies must disclose on these points even when the disclosure is negative.

Sample size	two cases report, N/A
Data exclusions	No data were excluded from the analysis
Replication	N/A
Randomization	N/A
Blinding	N/A - this is an open label compassionate use pilot study. Participants were blind in respect to the latency the effect of the compound starts after initiation of therapy. The participants were blind in respond to the duration of action of the compound after complete washout.

Reporting for specific materials, systems and methods

We require information from authors about some types of materials, experimental systems and methods used in many studies. Here, indicate whether each material, system or method listed is relevant to your study. If you are not sure if a list item applies to your research, read the appropriate section before selecting a response.

Materials & experimental systems

n/a	Involved in the study	
☒	☐	Antibodies
☒	☐	Eukaryotic cell lines
☒	☐	Palaeontology and archaeology
☒	☐	Animals and other organisms
☐	☒	Clinical data
☒	☐	Dual use research of concern
☒	☐	Plants

Methods

n/a	Involved in the study	
☒	☐	ChIP-seq
☒	☐	Flow cytometry
☒	☐	MRI-based neuroimaging

Clinical data

Policy information about clinical studies

All manuscripts should comply with the ICMJEguidelines for publication of clinical research and a completedCONSORT checklist must be included with all submissions.

Clinical trial registration	N/A pilot trial individual case of off-label use (compassionate use)
Study protocol	N/A - partially according to the REMPET study protocol
Data collection	Data collection was performed by the principal investigator. Images were collected at the respective Departments of Nuclear Medicine in Groningen and Amsterdam, The Netherlands and in Marburg, Germany.
Outcomes	Three outcome measures were employed: clinical outcome measure - the severity of the iRBD phenotype, two objective imaging methods: DAT SPECT and FDG PET. No difference was made between primary and secondary outcome measures.

Plants

Seed stocks	N/A
Novel plant genotypes	N/A
Authentication	N/A
